# Molecular Doping Mechanisms and Rational Molecular Design Strategies for High Doping Efficiency

**DOI:** 10.3390/polym18040501

**Published:** 2026-02-17

**Authors:** Hyojin Kye, Min Seon Kim, Bong-Gi Kim

**Affiliations:** 1 Department of Materials Science and Engineering, Konkuk University, Seoul 05029, Republic of Korea; hjk01109@konkuk.ac.kr; 2 Advanced Materials Program, Department of Materials Science and Engineering, Konkuk University, Seoul 05029, Republic of Korea

**Keywords:** molecular doping, conjugated polymer, F4TCNQ, organic thermoelectric

## Abstract

This review provides a comprehensive overview of molecular doping in organic semiconductors (OSCs), with particular emphasis on the mechanistic understanding of doping processes, rational material design strategies, and processing approaches for achieving high doping efficiency and stability. We discuss fundamental doping mechanisms, including integer charge transfer and orbital hybridization models, and highlight how molecular structure, polymer design, and dopant–host interactions influence electrical performance. Recent advances in processing strategies—such as sequential, vapor-phase, and hybrid doping methods—are also summarized in relation to microstructural control and charge transport optimization. In addition, the implications of molecular doping for emerging organic thermoelectric applications are addressed, emphasizing the interplay between dopant distribution, morphology, and device performance. By integrating mechanistic insights, material design principles, and application perspectives, this review aims to provide a unified framework for researchers in organic electronics, materials science, and thermoelectric device engineering seeking to develop highly efficient and stable molecularly doped organic conductors.

## 1. Introduction

The performance and stability of organic semiconductors (OSCs) have advanced remarkably over the past decades, largely driven by strategic molecular design and optimization of crystalline structures [1,2,3,4]. In particular, doping technologies for OSCs have recently attracted significant attention as an effective approach of enhancing electrical conductivity and charge-carrier density, thereby enabling their application in high-performance optoelectronic devices, such as organic field-effect transistors (OFETs) [5,6,7], organic light-emitting diodes (OLEDs) [8,9,10], photovoltaics [11,12,13], and organic thermoelectric generators [14,15,16]. There are two representative p-type doping strategies for OSCs: molecular doping and acid doping. Molecular doping employs strong electron acceptors, such as 2,3,5,6-tetrafluoro-7,7,8,8-tetracyanoquinodimethane (F4TCNQ), to induce charge transfer from electron-donating OSCs [17,18]. In contrast, acid doping relies on Lewis or Brønsted acids, which extract electrons from or protonate the conjugated backbone of OSCs [19,20].

Among doping approaches, molecular doping offers several advantages over acid-based doping. First, it provides superior chemical stability and compatibility, as molecular dopants are well-defined and less reactive than strong acids, minimizing undesired side reactions with OSCs or electrode materials [21]. Second, molecular doping preserves the structural integrity of the OSC backbone, whereas strong acids can cause irreversible chemical degradation through protonation or bond cleavage [22]. Third, molecular dopants offer tunability through molecular design; their electronic properties can be systematically adjusted via chemical modification (e.g., halogenation or side-chain engineering), enabling precise control over energy-level alignment and intermolecular interactions—capabilities that are difficult to achieve with inorganic or acid dopants [23,24,25]. Finally, molecular dopants typically exhibit good solubility in organic solvents, allowing facile solution processing and scalable fabrication, whereas acid dopants often require vapor-phase processing or suffer from poor solubility and aggregation issues [26,27]. Consequently, recent research has focused on improving doping efficiency by optimizing molecular doping mechanisms and developing new dopant structures with enhanced performance and stability [28,29,30,31,32,33,34].

**Figure 1:**
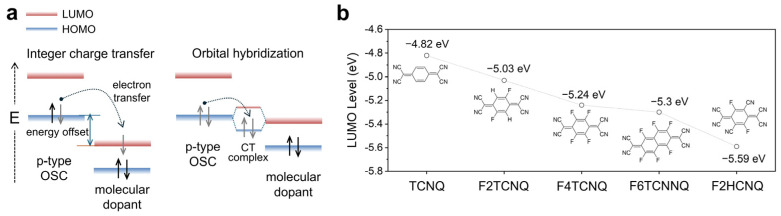
(**a**) Working principles of p-type molecular doping via integer charge transfer and orbital hybridization. (**b**) Evolution of the LUMO energy levels of TCNQ derivatives induced by the introduction of electron-withdrawing groups.

As illustrated in Figure 1a, the working principles of p-type molecular doping can be typically classified into two mechanisms. In the case of integer charge transfer (CT), dopants accept electrons directly from the highest occupied molecular orbital (HOMO) of electron-donating OSCs, leading to the formation of stabilized polarons and a significant increase in electrical conductivity [35,36,37]. In contrast, partial CT can occur through the formation of CT complexes arising from molecular orbital hybridization between the OSC and the dopant, particularly when the energy level offset is small [38,39,40]. However, integer CT generally yields a higher carrier density than CT complex formation, resulting in superior electrical conductivity [25,41].

From a theoretical perspective, achieving integer CT requires a sufficient energy offset between the HOMO of the OSC and the lowest unoccupied molecular orbital (LUMO) of the dopant, which serves as the driving force for spontaneous electron transfer from the OSC to the dopant. Accordingly, one strategy for developing molecular dopants has focused on deepening the LUMO level [25,42,43]. For example, tetracyanoquinodimethane (TCNQ) has been widely adopted as a core scaffold for molecular dopants, and the introduction of strong electron-withdrawing groups such as fluorine or nitrile moieties has been extensively explored to further lower the LUMO level (Figure 1b) [23,44].

Furthermore, among the various molecular dopants reported for OSCs, F4TCNQ has been most extensively investigated and is therefore frequently discussed in this review. This prominence arises from its relatively high electron affinity (~5.2 eV), good solubility in common organic solvents, and broad compatibility with a wide range of OSCs, which together enable efficient and reproducible molecular doping. As a result, F4TCNQ has become a benchmark dopant for elucidating fundamental molecular doping mechanisms, structure–property relationships, and processing effects in organic semiconductor systems.

Although several excellent review articles have addressed molecular doping in OSCs, most have primarily focused either on general doping mechanisms, dopant development, or device-oriented performance aspects [41,42]. In contrast, this review aims to comprehensively examine recent advances in p-type molecular doping and to highlight key structure–property relationships that govern the doping behavior of OSCs. Based on recent experimental and theoretical studies, we analyze how the chemical structures of both dopants and host OSCs influence CT efficiency, stability, and morphological compatibility. Special attention is given to the interplay between OSC structure, dopant energetics, and processing conditions, which collectively determine doping efficiency and electron transfer characteristics. Furthermore, we discuss the impact of processing parameters—such as solvent selection, thermal annealing, and doping methods (e.g., sequential versus co-doping)—on film morphology, phase separation, and doping homogeneity, as these factors play a critical role in determining the electrical performance of doped films. Finally, we outline strategic approaches to enhancing organic thermoelectric performance through molecular doping of OSCs. Collectively, these insights provide a foundation for advancing high-performance and stable organic conductors for electronic device applications through molecular-level engineering.

### Literature Search Strategy and Selection Criteria

To provide a comprehensive overview of molecular doping in OSCs, a systematic literature survey was conducted using major scientific databases, including Web of Science, Scopus, and Google Scholar. The literature search employed combinations of relevant keywords such as molecular doping, conjugated polymer doping, organic thermoelectrics, charge-transfer doping, and material design strategies for doping efficiency.

Articles were selected based on their relevance to (i) fundamental molecular doping mechanisms; (ii) molecular and polymer design strategies influencing doping efficiency; (iii) reporting of electrical, structural, or thermoelectric performance related to molecular doping. Particular emphasis was placed on experimentally validated studies; accordingly, this review primarily covers literature reporting experimental results rather than purely theoretical investigations. Priority was given to seminal studies that established key concepts, highly cited papers with significant impact in the field, and recent publications reflecting current research trends. Studies focusing primarily on acid-type doping, such as Lewis or Brønsted acid doping, as well as electrochemical doping, were excluded to maintain a clear focus on molecular doping systems.

The literature review primarily focuses on research published within approximately the past 10 years to capture recent developments, while earlier foundational works were included where necessary to provide historical context and conceptual background. This approach was adopted to ensure a balanced and representative discussion of molecular doping mechanisms, structure–property relationships, and emerging applications.

## 2. Molecular Doping and Mechanism

As discussed above, the energy offset between the HOMO of the OSC and the LUMO of the molecular dopant is a fundamental requirement for electron transfer during molecular doping. However, doping efficiency is not solely governed by the energy level alignment between the OSC and the dopant. In this section, we provide a comprehensive overview of the key factors that control molecular doping efficiency.

### 2.1. Energetic Requirements

**Figure 2:**
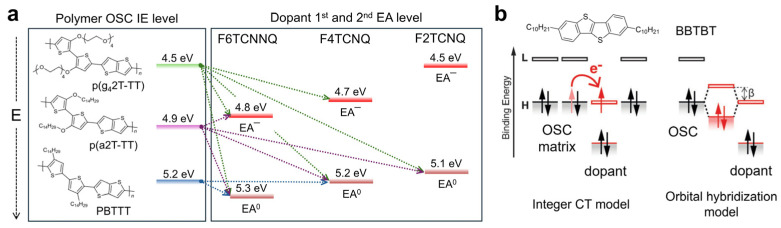
(**a**) Energy-level diagram illustrating dopant anion and dianion formation in p(g_4_2T-TT), p(a2T-TT), and pBTTT upon doping with F6TCNNQ, F4TCNQ, and F2TCNQ. (**b**) Relationship between the dopant EA and the doping efficiency of BBTBT, illustrating the impact of orbital hybridization (*β*) on the formation of ionized complexes. Reproduced with permission from Ref. [36]. Copyright 2013, Wiley-VCH GmbH.

Kiefer et al. demonstrated the importance of energy level offset by systematically controlling the ionization energy (IE) of OSCs and the electron affinity (EA) of dopants [45]. In this study, IE and EA correspond to the absolute energies of the HOMO and LUMO, respectively, and doping efficiency was directly quantified by analyzing the dopant anions formed after doping. When the IE of the conjugated polymer (CP, polymeric OSC) was lower than the EA (EA^0^) of the dopant employed, dopant anion formation was readily observed during the first doping step. Here, the electron affinity of the generated dopant anion (EA^−^) becomes lower than the initial electron affinity (EA^0^). If the IE of the CP remains lower than the EA^−^ of the dopant anion, the dopant anion was confirmed to participate in additional doping processes. For example, doping of P(g_4_2T-TT) with F2TCNQ resulted only in monoanion formation, whereas doping with F6TCNNQ readily led to the formation of dianions. In contrast, when p(a2T-TT) was doped with F4TCNQ, only F4TCNQ monoanions were observed, as the energy offset between the IE of p(a2T-TT) and the EA^−^ of F4TCNQ was insufficient to stabilize dianion formation (Figure 2a). Consequently, dopants capable of participating in two CT events formed dianions, resulting in ionization efficiencies of up to 200%. Although anion and dianion formation is influenced not only by energy level alignment but also by electrostatic interactions [46,47], these results clearly demonstrate the critical importance of the energy offset between the IE of the OSC and the EA of the dopant in achieving high doping efficiency.

### 2.2. Molecular Level Interactions

As discussed above, the conventional model of molecular doping in OSCs assumes integer CT from the HOMO of the OSC to the LUMO of the dopant, producing a localized dopant anion and a mobile polaron in the OSC. Accordingly, a high EA of the dopant is considered essential for efficient doping. In contrast, a recent alternative model describes molecular doping as arising from frontier molecular orbital hybridization between the OSC and the dopant, forming a ground-state CT complex when the energy offset between the two species is marginal [48,49,50,51]. Within this framework, doping efficiency is governed by the orbital energy splitting, quantified by the resonance integral *β*, which depends on orbital shape and relative orientation. Consistent with this model, Méndez et al. investigated the doping efficiency within the orbital hybridization framework using 2,7-didecyl[1]benzothieno-[3,2-b][1]benzothiophene (BBTBT) by varying the EA of fluorinated TCNQ dopants (Figure 2b), and demonstrated that minimizing the hybridization parameter *β* is crucial for enhancing doping efficiency by increasing the population of ionized complexes at room temperature [36].

**Figure 3:**
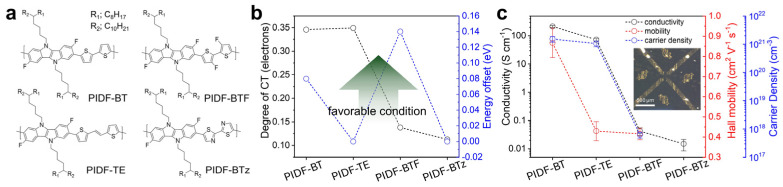
(**a**) Molecular structures of the IDID-based CPs. (**b**) Comparison of the degree of CT and the corresponding energy offset for four F4TCNQ-doped CP films. (**c**) Conductivity, Hall mobility, and carrier density of CP films after sequential F4TCNQ doping, determined by AC Hall effect measurements. Reproduced with permission from Ref. [52]. Copyright 2020, Wiley-VCH GmbH.

As another example highlighting the effect of molecular level interaction in enhancing doping efficiency, Park et al. systematically compared CPs incorporating an electron donating indoloindole (IDID) unit with different comonomers to tune their HOMO energy level (Figure 3a) [52]. By analyzing the CT interactions, they found that the degree of CT between the CPs and F4TCNQ showed no clear correlation with the energy offset between them (Figure 3b). Furthermore, direct quantification of electrical properties using AC Hall effect measurements revealed that the carrier density closely followed the trend of CT degree rather than that of the energy offset (Figure 3c). These results clearly demonstrate that, while energy level alignment is a prerequisite for molecular doping, doping efficiency is more sensitively governed by the strength of CT interactions between the OSC and the dopant.

## 3. Doping Method for High Doping Efficiency

Electrical conductivity is governed by both carrier density and charge-carrier mobility, indicating that maintaining high mobility is as critical as increasing carrier density upon doping. Owing to their strong electron-donating character and inherently high mobility, thiophene-based OSCs are among the most widely used platforms for molecular doping [53,54,55,56,57,58,59,60,61]. Because high mobility in OSCs is closely linked to crystalline morphology [62,63,64,65], simple blending of dopants often degrades crystallinity and reduces mobility as dopant loading increases [66,67,68]. Consequently, advanced doping strategies have been developed to increase dopant concentration while preserving crystalline order, thereby enhancing overall electrical conductivity

### 3.1. Direct Blend Doping

**Figure 4:**
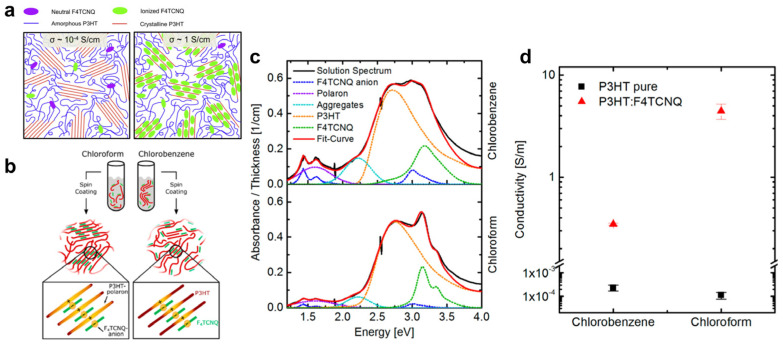
(**a**) Schematic illustration showing the dopant concentration-dependent doping behavior of P3HT with F4TCNQ (right: low concentration; left: high concentration). Reproduced with permission from Ref. [69]. Copyright 2013, Elsevier. (**b**) Schematic depiction of the effects of solvent polarity on the solubility of the CT complex and the resulting film morphology, comparing polar CF with CB. (**c**) UV–vis absorption spectra of P3HT:F4TCNQ in CB (top) and CF (bottom). (**d**) Lateral conductivity of P3HT thin films processed from CB and CF. Reproduced with permission from Ref. [74]. Copyright 2016, American Chemical Society.

Direct blending of OSCs with molecular dopants in solution is the simplest approach to induce molecular doping. Duong et al. investigated the relationship between doping efficiency and dopant concentration by blending poly(3-hexylthiophene) (P3HT) with F4TCNQ [69]. They showed that the nature of the dopant–P3HT interaction strongly depends on dopant content. At low dopant concentrations (weak doping regime), neutral F4TCNQ and P3HT coexist with low ionization efficiency. At intermediate concentrations (strong doping regime), dopant–P3HT complexes form in solution, promoting co-crystalline phases and leading to a pronounced increase in electrical conductivity (Figure 4a). In contrast, further increasing the dopant content results in reduced ionization efficiency and conductivity due to dopant saturation. These concentration-dependent morphological and electrical changes were later directly visualized by conductive atomic force microscopy (c-AFM) in follow-up studies [70].

However, direct solution blending of an OSC with a molecular dopant often induces CT complex formation, leading to precipitation because the solubility of the complex change drastically [71,72,73]. Consequently, the solubility of the charged CT complex is a critical parameter for optimizing doping efficiency in blend-doped systems. Müller et al. showed that chloroform (CF) enables more efficient doping of P3HT films than chlorobenzene (CB), attributing this to the higher solubility of the charged complex (P3HT^+^/F4TCNQ^−^) in the more polar CF. In contrast, strong pre-aggregation in CB hinders film crystallinity and interdomain connectivity (Figure 4b) [74]. UV–Vis absorption spectra further revealed that P3HT:F4TCNQ solutions in CB exhibit stronger aggregate-, F4TCNQ anion-, and polaron-related features than those in CF (Figure 4c), indicating the formation of pre-aggregated domains in solution. As a result, films processed from CF show approximately an order-of-magnitude higher electrical conductivity than those from CB, owing to their more homogeneous and well-ordered morphology (Figure 4d).

**Figure 5:**
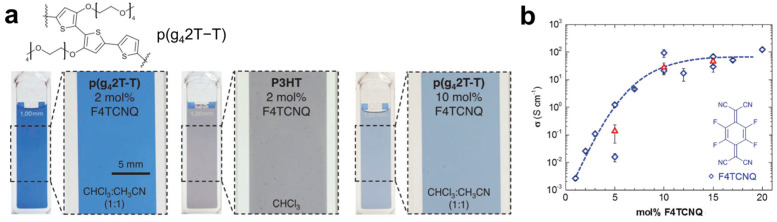
(**a**) Photographs of p(g_4_2T-T) and P3HT solutions doped with F4TCNQ at different dopant concentrations and in different solvents. (**b**) Electrical conductivity (σ) as a function of F4TCNQ molar fraction. Reproduced with permission from Ref. [75]. Copyright 2017, Wiley-VCH GmbH.

Beyond solvent selection, incorporating polar substituents into the OSC backbone is an effective strategy to enhance both the solubility of charged complexes in solution and dopant–OSC miscibility in the solid state. Kroon et al. demonstrated this approach by engineering polythiophene with tetraethylene glycol side chains (p(g_4_2T-T)) [75]. These polar side chains improve the solubility of ionized complexes in CF and strengthen interactions with F4TCNQ anions (Figure 5a), yielding films with electrical conductivities up to 100 S·cm^−1^ and excellent thermal stability up to 150 °C (Figure 5b).

### 3.2. Sequential Solution Doping

An alternative strategy for producing high-quality doped films is sequential doping, which avoids CT reactions in solution. In this approach, a pre-formed OSC film is either immersed in a dopant solution or coated with the dopant in a subsequent deposition step, allowing dopant diffusion into the OSC matrix while preserving the original film morphology [76,77,78]. For example, Scholes et al. applied sequential doping by spin-coating F4TCNQ from a tetrahydrofuran (THF)/dichloromethane (DCM) solution onto pre-formed P3HT films [73]. Compared with the conductivity (<1.0 S·cm^−1^) obtained from doped blend films prepared from mixed solutions, the sequentially doped films exhibited significantly higher electrical conductivity (5.5 S·cm^−1^) while maintaining an undisturbed morphology.

**Figure 6:**
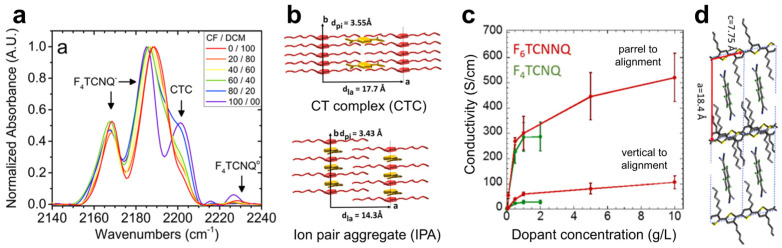
(**a**) UV/Vis absorption spectra of F4TCNQ-doped P3HT film with varying CF/DCM ratios. (**b**) Schematic illustration of CT complex (CTC, top) and ion-pair aggregation (IPA, bottom) formation in F4TCNQ-doped P3HT, showing the incorporation of F4TCNQ within the unit cell. Reproduced with permission from Ref. [71]. Copyright 2016, American Chemical Society. (**c**) Electrical conductivity of oriented semicrystalline P3HT films doped with F4TCNQ (green) and F6TCNNQ (dark red). (**d**) Packing structure of P3HT-F6TCNNQ. Reproduced with permission from Ref. [79]. Copyright 2016, American Chemical Society.

Stanfield et al. tuned the microstructure of sequentially doped P3HT films by selecting appropriate casting solvents [71]. The good solvent CF partially dissolves P3HT, facilitating incorporation of F4TCNQ anions into the polymer π-stacks, whereas the semi-orthogonal solvent DCM selectively swells the alkyl side chains without disrupting the backbone. UV–Vis absorption analysis as a function of solvent blend ratio shows that CF promotes charge-transfer complex (CTC) formation via dopant intercalation into π–π stacking regions (Figure 6a), while DCM favors ion-pair aggregate (IPA) formation through incorporation into lamellar domains (Figure 6b). Consequently, DCM-treated films exhibit higher electrical conductivity (>5.5 S·cm^−1^) than CF-treated films (>2.5 S·cm^−1^), consistent with the integer CT nature of the IPA state.

As discussed above, one of key advantages of sequential doping is enhancing doping efficiency while preserving charge-carrier mobility by enabling dopant diffusion through amorphous regions without disrupting the optimal high-mobility morphology. Recently, Untilova et al. fabricated uniaxially aligned regio-regular P3HT films and applied sequential doping to probe charge transport along directions parallel and perpendicular to the polymer alignment [79]. As shown in Figure 6c, both F4TCNQ- and F6TCNNQ-doped films exhibited electrical conductivities more than five times higher along the alignment direction than in the perpendicular direction, demonstrating that sequential doping preserves the intrinsic morphology of the pre-deposited P3HT films. Moreover, transmission electron microscopy (TEM) and polarized absorption spectroscopy revealed that F6TCNNQ achieves a higher degree of ordering within P3HT crystals than F4TCNQ, resulting in enhanced electrical conductivity of up to 500 S·cm^−1^ (Figure 6d).

### 3.3. Sequential Vapor Doping

Sequential doping using dopant solutions has been widely studied; however, the morphology of pre-deposited films can be compromised when the OSC lacks sufficient orthogonal solubility in the dopant solution. To overcome this limitation, sequential vapor doping, which introduces dopants into pre-deposited OSC films via thermal evaporation, has been proposed [30,80,81]. For example, Kang et al. incorporated F4TCNQ into the highly ordered lamellar microstructure of regioregular poly(2,5-bis(3-hexadecylthiophen-2-yl)thieno[3,2-b]thiophene) (PBTTT) through solid-state thermal diffusion [82]. The side chains of PBTTT exhibit strong compatibility with F4TCNQ, enabling efficient dopant diffusion into the solid film. As a result, sequential vapor doping of PBTTT yields coherent, free-electron-like charge transport and high electrical conductivity of up to 248 S·cm^−1^.

**Figure 7:**
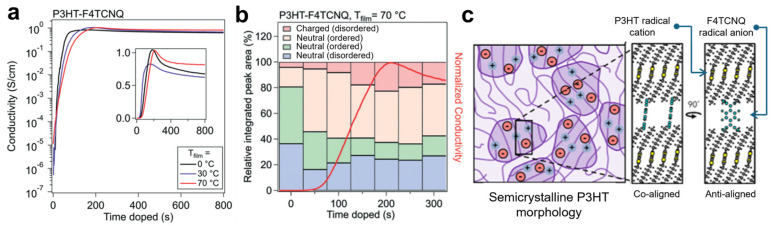
(**a**) In situ conductivity curves of F4TCNQ-doped P3HT at three different temperatures. Inset: semi-log plot. (**b**) Integrated peak areas obtained from Raman peak fitting of P3HT–F4TCNQ samples at a film temperature (T_film) of 70 °C. Reproduced with permission from Ref. [83]. Copyright 2022, Royal Society of Chemistry. (**c**) Schematic depiction of hypothetical doping distributions, illustrating the alignment of F4TCNQ radical anion relative to P3HT radical cation within crystalline domains. Reproduced with permission from Ref. [84]. Copyright 2024, American Chemical Society.

DiTusa et al. used in situ conductivity measurements to track the conductivity evolution of P3HT thin films during F4TCNQ vapor doping at different temperatures [83]. Conductivity showed a rapid exponential rise, followed by a brief linear increase to a maximum, and then a slow decay at all temperatures (Figure 7a). Raman spectroscopy further revealed that this evolution correlates with the progression of doping in both crystalline (ordered) and amorphous (disordered) P3HT domains (Figure 7b). Uniform dopant distribution is also critical for maximizing doping efficiency and achieving high conductivity in sequential doping systems. Recently, Nguyen et al. employed polarized resonant soft X-ray scattering (P-RSoXS) to probe counterion distribution in doped P3HT films with systematically varied crystallinity [84]. By blending regio-regular and regio-random P3HT and infiltrating F4TCNQ via thermal evaporation, they found that P-RSoXS anisotropy indicates an anti-aligned configuration in crystalline domains, with the conjugated planes of F4TCNQ oriented perpendicular to those of P3HT rather than coaligned (Figure 7c).

### 3.4. Hybrid Doping

Sequential doping preserves the optimal morphology of pre-deposited OSCs; however, dopant diffusion can be severely hindered in tightly packed crystalline domains, limiting achievable dopant loading [85,86]. To overcome this limitation, a hybrid doping strategy that combines blend and sequential doping has been proposed. As discussed above, molecular dopants can strongly interact with electron-donating OSCs and form aggregates in solution at high concentrations, whereas below a critical concentration the dopant–OSC interaction remains weak and both species are uniformly dissolved. Films cast from such dilute blends allow dopants to be incorporated directly into the conjugated units of OSCs, slightly expanding the π–π stacking distance within crystalline domains. Subsequent sequential doping then facilitates efficient dopant diffusion and increases dopant loading via redistribution of the pre-embedded dopants, ultimately maximizing electrical conductivity.

**Figure 8:**
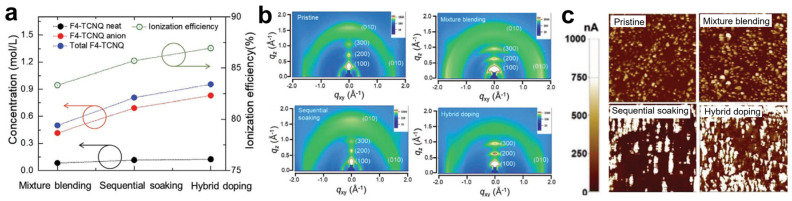
(**a**) Composition and ionization efficiency of P3HT films doped via mixture blending, sequential soaking, and hybrid doping processes. (Red and black arrows: concentration; green arrows: ionization efficiency). (**b**) 2D GIXD patterns of pristine P3HT films and doped P3HT films prepared using different doping methods. (**c**) Current mapping images of pristine and doped P3HT films obtained by the different doping processes. Reproduced with permission from Ref. [87]. Copyright 2020, Wiley-VCH GmbH.

Yoon et al. compared the doping efficiency of blend, sequential, and hybrid doping strategies using P3HT [87]. Quantification of F4TCNQ anions by UV–Vis absorption revealed an increasing dopant concentration in the order of blend, sequential, and hybrid doping (Figure 8a). Two-dimensional grazing-incidence X-ray diffraction (2D GIXRD) showed that blend doping enhanced P3HT crystallinity relative to pristine films due to strong dopant–polymer interactions, whereas sequential doping slightly reduced crystallinity because of film swelling at high dopant loadings. In contrast, hybrid doping yielded increased crystallinity compared with pristine and sequentially doped films, albeit to a lesser extent than blend doping (Figure 8b). c-AFM further demonstrated that hybrid doping produced broader and more uniformly distributed highly conductive regions than the other strategies (Figure 8c). Consequently, electrical conductivity increased from 1.4 S·cm^−1^ (blend) to 26.0 S·cm^−1^ (sequential) and 71.2 S·cm^−1^ (hybrid). These results indicate that hybrid doping synergistically combines the advantages of sequential doping—higher carrier density via increased dopant loading—and blend doping—enhanced mobility through improved crystallinity.

## 4. OSC Design Strategies for High Doping Efficiency

The efficiency of molecular doping is closely linked to the electron-donating properties of OSCs, while electrical conductivity is determined by both the carrier density and mobility achieved upon doping. Accordingly, thiophene and its derivatives, which offer strong electron-donating character and relatively high charge mobility, have been extensively explored as molecular doping platforms. Notably, polythiophene derivatives such as PBTTT have achieved electrical conductivities exceeding 200 S·cm^−1^ after molecular doping, owing to their high intrinsic mobility and efficient doping characteristics [30,59,60]. In this section, we review representative recent examples of molecular doping in polythiophenes, with emphasis on conjugated backbone engineering to simultaneously enhance doping efficiency and mobility, as well as side-chain engineering strategies that facilitate dopant diffusion during sequential doping.

### 4.1. Conjugated Frame Engineering

**Figure 9:**
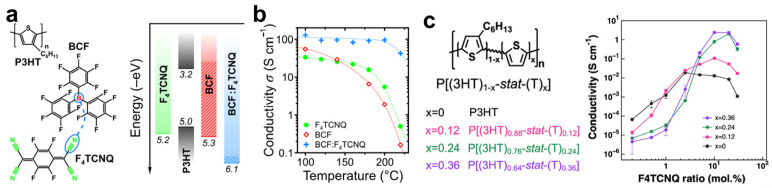
(**a**) Chemical structures of P3HT doped with BCF:F4TCNQ complexes, along with the corresponding energy levels of P3HT and the dopants. (**b**) Electrical conductivity of doped P3HT films after sequential annealing at increasing temperatures. Reproduced with permission from Ref. [55]. Copyright 2024, American Chemical Society. (**c**) Molecular structures of P3HT and its three random polymers, and the electrical conductivity of the corresponding doped polymer films as a function of dopant concentration. Reproduced with permission from Ref. [18]. Copyright 2023, Elsevier.

Although P3HT is a strong electron-donating OSC, its pronounced crystallinity suppresses dopant diffusion, resulting in limited molecular doping efficiency. To address this limitation, strategies based on dopant system engineering and polymer structural modification have been explored. For example, Zapata-Arteaga et al. designed Lewis-paired dopant complexes by combining the Lewis acid B(C_6_F_5_)_3_ (BCF) with F4TCNQ [55]. Owing to the higher electron affinity of the BCF:F4TCNQ complex relative to the individual components (Figure 9a), P3HT films doped with this complex achieved conductivities exceeding 300 and 900 S·cm^−1^ for isotropic and chain-oriented films, respectively. These values represent the highest reported conductivities for doped P3HT, and the BCF:F4TCNQ system also exhibited a threefold higher thermal dedoping activation energy than neat dopants, ensuring stable doping above 200 °C (Figure 9b). In a complementary approach, Kim et al. synthesized random copolymers of 3-hexylthiophene and thiophene (P[(3HT)_1-x_−stat–(T)_x_]) to mitigate mobility loss associated with microstructural collapse at high dopant loadings [18]. For compositions with x ≥ 0.24, the F4TCNQ-doped copolymers exhibited electrical conductivities more than two orders of magnitude higher than those of F4TCNQ-doped P3HT homopolymer at an F4TCNQ ratio of approximately 10 mol% (Figure 9c).

**Figure 10:**
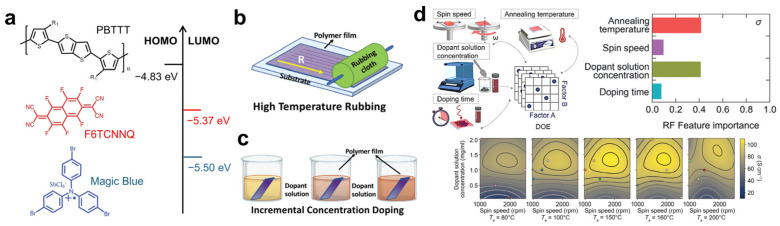
(**a**) Chemical structures of PBTTT polymers and dopants, along with their corresponding energy levels. (**b**) Schematic illustration of the fabrication process for oriented PBTTT films using high-temperature rubbing. (**c**) Sequential doping procedure achieved through incremental increases in dopant concentration. Reproduced with permission from Ref. [60]. Copyright 2013, Wiley-VCH GmbH. (**d**) Schematic overview of ML-assisted process optimization for high-performance OTE devices, illustrating correlations between process factors and electrical conductivity (σ). Correlations between TE properties and σ are presented as 2D contour plots at specific annealing temperatures (*T_a_*). Reproduced with permission from Ref. [88]. Copyright 2024, Wiley-VCH GmbH.

Recently, Guchait et al. employed oriented PBTTT films to maximize electrical conductivity through molecular doping [60]. The electrical conductivities of PBTTT doped with F6TCNNQ and the Lewis acid dopant Magic Blue—both possessing deeper LUMO levels than the HOMO of PBTTT—were directly compared (Figure 10a). Exploiting the liquid-crystalline behavior of PBTTT at elevated temperatures, chain alignment was achieved via a high-temperature rubbing process (Figure 10b), and the degree of orientation was quantified using TEM, polarized UV–vis–NIR spectroscopy, and charge transport measurements. Notably, a sequential doping strategy involving a gradual increase in dopant concentration was shown to maximize doping efficiency while minimizing microstructural disruption of PBTTT (Figure 10c). As a result, F6TCNNQ-doped, oriented PBTTT films exhibited an electrical conductivity of up to 2400 S·cm^−1^, approximately 18 times higher than that of randomly oriented films (134 S·cm^−1^). Consistently, Dash et al. reported conductivities exceeding 2400 S·cm^−1^ in similarly oriented PBTTT films doped with F6TCNNQ [63].

Meanwhile, Jeong et al. proposed a machine learning-based optimization of the sequential doping process for PBTTT [88]. Using spin-coating speed, annealing temperature, dopant solution concentration, and doping time as input parameters, feature importance was evaluated via a random forest algorithm. Among these parameters, annealing temperature and dopant solution concentration were identified as the most influential factors governing post-doping electrical conductivity (Figure 10d).

**Figure 11:**
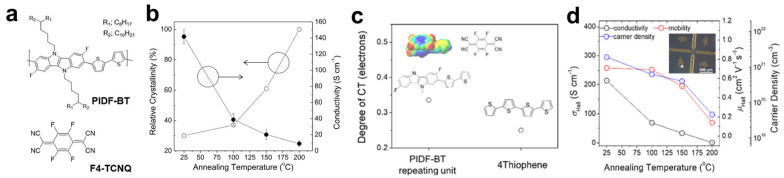
(**a**) Chemical structures of PIDF-BT and F4TCNQ. (**b**) Relationship between the relative crystallinity and electrical conductivity of F4TCNQ-doped PIDF-BT films under different annealing temperatures. The arrows indicate the corresponding y-axis. Reproduced with permission from Ref. [85]. Copyright 2019, Elsevier. (**c**) Calculated CT degree of PIDF-BT compared with 4 thiophene in the presence of F4TCNQ. (**d**) Annealing temperature dependent four-probe conductivity, Hall mobility, and carrier density of doped PIDF-BT films. Reproduced with permission from Ref. [86]. Copyright 2019, American Chemical Society.

The high molecular doping efficiency of polythiophenes arises from their strong electron-donating character and high charge mobility; accordingly, incorporating highly electron-donating moieties into polythiophene backbones has been widely adopted to enhance doping efficiency in OSCs. For example, Park et al. investigated F4TCNQ doping in PIDF-BT, a copolymer comprising the electron-donating IDID unit and bithiophene (Figure 11a) [85]. Although thermal annealing increased PIDF-BT crystallinity, it impeded dopant diffusion during sequential doping, leading to reduced doping efficiency. Consequently, high electrical conductivity (>140 S·cm^−1^) was achieved when doping was performed without prior film annealing (Figure 11b). In a follow-up study, Yoon et al. attributed the superior doping behavior of PIDF-BT to stronger CT interactions with F4TCNQ, enabled by the enhanced electron-donating nature of the IDID unit (Figure 11c) [86]. AC Hall measurements further revealed that increased crystallinity suppresses dopant diffusion, reduces carrier density, and ultimately lowers conductivity (Figure 11d). By optimizing the sequential doping conditions (e.g., annealing temperature and doping time), electrical conductivity exceeding 200 S·cm^−1^ was achieved in F4TCNQ-doped PIDF-BT.

**Figure 12:**
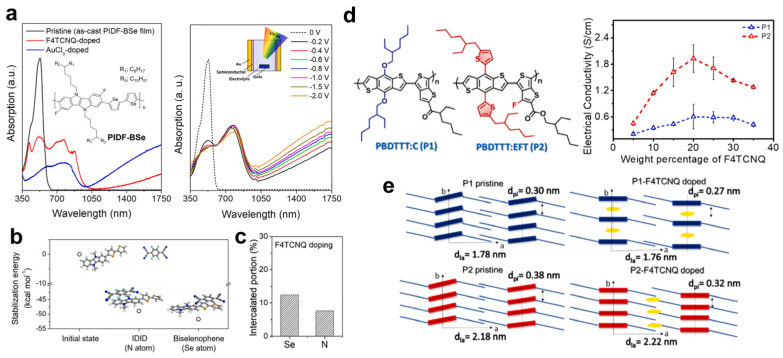
(**a**) Absorption spectra of pristine and chemically doped PIDF-BSe films with F4TCNQ and AuCl_3_, and the evolution of electrochemically doped spectra as a function of the applied gate voltage. (**b**) Stabilization energies of F4TCNQ interacting with IDID and selenophene units under optimized geometries. (**c**) Intercalated fractions of Se and N atoms in PIDF-BSe upon F4TCNQ doping, as determined by XPS. Reproduced with permission from Ref. [89]. Copyright 2022, Elsevier. (**d**) Molecular structures of polymers with alkyl and thiophene-based side chains and their electrical conductivity as a function of F4TCNQ content. (**e**) Illustration of polymer chain rearrangement induced by F4TCNQ doping. The arrows indicate the interplanar distance. Reproduced with permission from Ref. [90]. Copyright 2024, Elsevier.

Selenophene is theoretically a stronger electron donor than thiophene due to the incorporation of selenium into the five-membered aromatic ring. Accordingly, replacing thiophene with selenophene in conjugated backbones has been explored as a strategy to enhance the doping efficiency of organic semiconductors. Han et al. systematically investigated the doping behavior of PIDF-BSe, a copolymer of IDID and biselenophene, in which bithiophene was replaced by biselenophene [89]. By comparing the absorption changes induced by chemical and electrochemical doping, they identified spectral features associated with polaron and bipolaron formation in PIDF-BSe (Figure 12a). Density functional theory calculations further indicated that coupling between PIDF-BSe and F4TCNQ preferentially occurs at the biselenophene units rather than the IDID units (Figure 12b), which was corroborated by X-ray photoelectron spectroscopy showing stronger interaction of F4TCNQ with Se atoms than with N atoms in the IDID unit (Figure 12c). As a result, F4TCNQ-doped PIDF-BSe exhibited excellent electrical conductivity exceeding 200 S·cm^−1^.

The doping efficiency of fused aromatic thiophenes has been investigated to elucidate structure-dependent dopant interactions. Raveendran et al. designed two polymer OSCs sharing the same benzodithiophene–co–thieno[3,2-b]thiophene backbone but differing in side-chain structure [90]. Introducing an alkoxy side chain (BDTTT:C, P1) led to the formation of partial CT complexes with F4TCNQ, whereas incorporating a thiophene-based conjugated side chain (BDTTT:EFT, P2) favored integer CT complexes, resulting in more than a threefold increase in electrical conductivity (Figure 12d). GIXRD and high-resolution TEM revealed that F4TCNQ intercalates into the polymer π-stacks in P1, while in P2 it preferentially resides in the alkyl side-chain or lamellar regions (Figure 12e), consistent with prior reports by Stanfield et al. [71]. These findings indicate that integer CT states arise from dopant intercalation within lamellar domains, whereas partial CT states originate from dopant π-stacking within the conjugated backbone, where orbital overlap is maximized.

### 4.2. Side-Chain Engineering

In general, molecular dopants are more polar than electron-donating OSCs; therefore, introducing polar side chains into OSCs is an effective strategy to suppress phase separation and enhance dopant–OSC miscibility. In particular, ethylene glycol (EG) side chains have been widely explored as alternatives to hydrophobic alkyl chains to improve doping efficiency [75,91,92,93,94,95].

**Figure 13:**
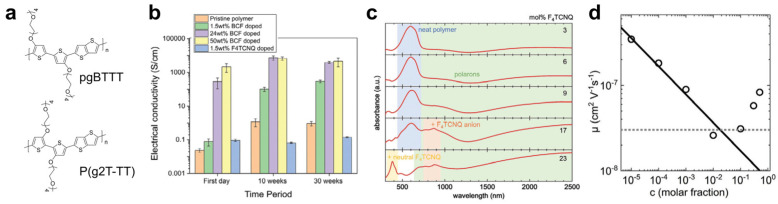
(**a**) Molecular structures of pgBTTT and P(g2T-TT) polymers. (**b**) Air stability of the electrical conductivity of pgBTTT over 10 and 30 weeks. Reproduced with permission from Ref. [91]. Copyright 2024, Wiley-VCH GmbH. (**c**) UV–vis–NIR spectra of F4TCNQ-doped p(g_4_2T-T) films at various doping concentrations. (**d**) Kinetic Monte Carlo simulation showing the dependence of charge-carrier mobility (μ) on the dopant concentration. Reproduced with permission from Ref. [96]. Copyright 2024, Wiley-VCH GmbH.

Chen et al. compared the doping behavior of PBTTT, p(g2T-TT), and pgBTTT—where the alkyl side chains of PBTTT were replaced with EG-based side chains—using F4TCNQ and the Lewis acid dopant BCF via blend doping (Figure 13a) [91]. Owing to improved dopant miscibility, the electrical conductivity for both dopants increased in the order of PBTTT < p(g2T-TT) < pgBTTT. Notably, pgBTTT exhibited an electrical conductivity exceeding 2000 S·cm^−1^, far higher than that of p(g2T-TT) (380 S·cm^−1^). This exceptional performance was attributed to the improved backbone planarity of pgBTTT and the increased accessibility of dopants arising from the distinct positioning of the EG-based side chains. In addition, EG substitution significantly enhanced doping stability, with the electrical conductivity of pgBTTT remaining stable or slightly increasing over 30 weeks (Figure 13b).

Liu et al. systematically investigated the doping behavior of conjugated polymers bearing EG-based side chains using p(g_4_2T-T) and F4TCNQ blend solutions (structure shown above Figure 5a) [96]. Owing to the EG substitution, p(g_4_2T-T) exhibited excellent miscibility with F4TCNQ up to 23 mol%, leading to an increase in electrical conductivity from 0.4 to 72.3 S·cm^−1^ as the dopant concentration increased from 3 to 23 mol%. UV–vis–NIR spectra showed a progressive decrease of the ~600 nm absorption associated with the neutral polymer and the emergence of polaronic absorption in the near-infrared, indicating strong doping at high dopant loadings (Figure 13c). At 17 and 23 mol% F4TCNQ, additional absorption features at 800–900 nm, characteristic of F4TCNQ anions, were observed, while films doped at 3–9 mol% predominantly contained F4TCNQ dianions. In contrast, charge-carrier mobility decreased with increasing dopant concentration due to enhanced energetic disorder and charge trapping by ionized dopants, which outweigh the benefits of state filling, consistent with previous reports (Figure 13d) [96,97].

**Figure 14:**
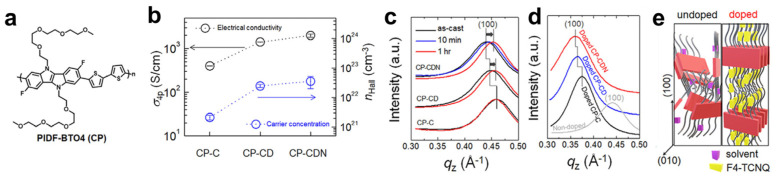
(**a**) Molecular structure of PIDF-BTO4 (CP). (**b**) Electrical conductivity and charge-carrier density of F4TCNQ-doped PIDF-BTO4 films prepared using different processing solvents. Black arrows indicate *σ_4p,_* and blue arrows indicate *n_Hall_*. (**c**,**d**) Out-of-plane (100) diffraction patterns of PIDF-BTO4 films processed from different solvents: (**c**) pristine (non-doped) films as a function of drying time and (**d**) doped PIDF-BTO4 films. (**e**) Schematic illustration depicting changes in the molecular arrangement of PIDF-BTO4 chains upon doping. Reproduced with permission from Ref. [98]. Copyright 2023, Cell Press.

Similar to the role of EG-based side chains in promoting dopant diffusion, controlling the volatility of processing solvents has emerged as an effective strategy to enhance doping efficiency in CP films. Yoon et al. synthesized PIDF-BTO4 by introducing EG-based side chains into PIDF-BT and investigated the effect of solvent volatility on molecular doping (Figure 14a) [98]. Single (CF), binary (CF:o-dichlorobenzene, DCB), and ternary (CF:DCB:N-methylaniline, NMA) solvent systems were employed, selected based on polymer solubility and decreasing volatility in the order CF > DCB > NMA. Following F4TCNQ doping, electrical conductivity increased systematically from 400.8 S·cm^−1^ (CP-C) to 1416.9 S·cm^−1^ (CP-CD) and 2003.2 S·cm^−1^ (CP-CDN), consistent with an increased charge-carrier density (Figure 14b). Two-dimensional GIXD revealed that residual solvent in CP-CD and CP-CDN facilitates polymer chain rearrangement (Figure 14c), while subsequent sequential doping induces tighter π–π stacking and enhanced crystallinity relative to pristine films (Figure 14d). Overall, enhanced diffusion of F4TCNQ promotes a molecular assembly transition from mixed to edge-on-dominant orientations in PIDF-BTO4, leading to backbone planarization, improved carrier mobility, and ultimately higher electrical conductivity.

## 5. Organic Thermoelectric Applications

**Figure 15:**
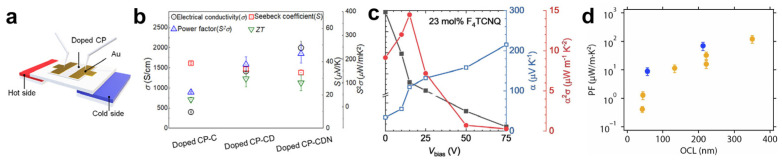
(**a**) Schematic diagram illustrating thermoelectric measurements of doped CPs. (**b**) Thermoelectric properties of doped CP-C, CP-CD, and CP-CDN films. Reproduced with permission from Ref. [98]. Copyright 2023, Cell Press. (**c**) Thermoelectric properties obtained under different applied bias voltages for doped p(g_4_2T-T) films with 23 mol% F4TCNQ. Reproduced with permission from Ref. [96]. Copyright 2024, Wiley-VCH GmbH. (**d**) Relationship between the orientational correlation length (OCL) and power factors (PF) (Yellow: F4TCNQ; Blue: F2TCNQ). Reproduced with permission from Ref. [47]. Copyright 2017, AAAS.

Organic thermoelectric (OTE) materials offer inherent advantages, including mechanical flexibility, low thermal conductivity, light weight, and solution processability. Among them, doped CPs have attracted considerable attention due to their tunable electronic properties and compatibility with scalable fabrication. The thermoelectric effect describes the generation of a thermovoltage in response to a temperature gradient across a material (Figure 15a), and thermoelectric performance is quantified by the dimensionless figure of merit, *ZT* = *S*2*σT*/*κ*, where *S* is the Seebeck coefficient, *σ* the electrical conductivity, *T* the absolute temperature, and *κ* the thermal conductivity. Because the thermal conductivity of organic materials typically varies within a narrow range, the power factor (*S*2*σ*) is often used as a practical metric to evaluate OTE performance.

CPs inherently possess low thermal conductivity, which is advantageous for thermoelectrics, but their electrical conductivity is typically insufficient. Molecular doping is therefore essential to increase charge-carrier density and electrical conductivity, enabling practical OTE performance. Most studies on doped CPs have focused on conductivity enhancement and the resulting OTE properties; accordingly, this section briefly reviews the thermoelectric performance of representative CPs discussed in the preceding doping strategies. For example, glycolated polymers such as PIDF-BTO4 demonstrate that EG-based side chains markedly enhance dopant diffusion and miscibility, leading to high electrical conductivity and power factors exceeding 260 μW·m^−1^·K^−2^ through controlled solution processing (Figure 15b) [98]. Similarly, p(g_4_2T-T), incorporating oligo(ethylene glycol) side chains, exhibits excellent miscibility with F4TCNQ at high dopant concentrations, enabling systematic tuning of carrier density and power factors up to 14.5 μW·m^−1^·K^−2^ via graded doping architectures (Figure 15c) [96].

In contrast, alkyl-substituted benchmark polymers such as PBTTT rely primarily on high intrinsic crystallinity and molecular ordering to achieve efficient charge transport upon F4TCNQ doping, and thus often serve as reference systems for high electrical conductivity and power factor. For example, vapor-doped PBTTT films with different orientational correlation lengths (OCLs) have achieved power factors of up to 120 μW·m^−1^·K^−2^ (Figure 15d) [47]. More recent studies further demonstrate that precise control over F4TCNQ diffusion and spatial distribution—rather than increased dopant loading alone—is crucial for balancing electrical conductivity and the Seebeck coefficient [30,99]. Collectively, these findings highlight that although F4TCNQ remains an effective p-type dopant, ultimate thermoelectric performance is governed by the interplay of dopant–polymer interactions, side-chain polarity, and microstructural order, with EG-based side chains offering a particularly promising route toward high and controllable thermoelectric efficiency.

## 6. Summary and Perspectives

Molecular doping is a powerful approach for enhancing the electrical properties of OSCs; however, achieving both high doping efficiency and long-term stability remains challenging. Increasing dopant concentration does not necessarily yield a higher density of mobile carriers due to incomplete CT, strong Coulomb binding between dopant ions and polarons, and dopant aggregation that disrupts the host’s microstructure. These factors often impose a trade-off between electrical conductivity and charge-carrier mobility, particularly in semicrystalline OSCs. For convenience, key molecular doping systems, doping strategies, and representative electrical performance metrics discussed in this review are summarized in Table 1.

Despite considerable progress, several fundamental aspects of molecular doping mechanisms remain under active debate. In particular, the relative importance of integer charge transfer versus orbital hybridization models has not been fully resolved. While integer CT is widely regarded as the dominant mechanism for generating mobile carriers, increasing evidence suggests that hybridization-induced charge-transfer complexes can significantly influence doping efficiency depending on energy-level alignment, molecular packing, and local microstructure. Resolving these discrepancies requires more systematic studies combining spectroscopic, structural, and theoretical analyses under comparable conditions.

Future progress requires moving beyond empirical dopant selection toward rational co-design of dopants and host polymers. Key strategies include engineering OSC backbones and side chains to balance dopant miscibility with crystallinity, tailoring dopant structures to optimize ionization energetics while suppressing aggregation, and employing processing routes—such as sequential, vapor-phase, or solid-state diffusion doping—to precisely control dopant diffusion and spatial distribution. Equally important is the development of standardized descriptors and correlative characterization methods that link dopant distribution and local electronic structure to macroscopic transport properties, enabling more reliable comparison across different material systems.

Looking ahead, several research priorities emerge. Improved control over dopant aggregation and spatial distribution will be essential to mitigate the trade-off between carrier density, mobility, and long-term stability. Scalable and reproducible processing strategies must also be developed to translate laboratory-scale advances into practical applications. Finally, improving environmental and thermal stability under operating conditions remains critical, particularly for emerging OTE applications. Addressing these challenges through an integrated dopant–host–process design framework is expected to enable the next generation of highly efficient and stable molecularly doped organic conductors and, ultimately, high-performance OTE generators.

## Figures and Tables

**Table 1:** Summary of representative molecular doping systems, doping strategies, and resulting electrical properties.

Doping Method	Materials		Conductivity	Ref.
	OSCs	Dopant		
Blend doping	P3HT	F4TCNQ	1.82 S/cm	[69]
	P(g_4_2T-TT)	F4TCNQ/F6TCNNQ	~100 S/cm	[45]
	P[(3HT)_0.64_-stat-(T)_0.36_]	F4TCNQ	2.4 S/cm	[18]
	P(g_4_2T-T)	F4TCNQ	~100 S/cm	[75]
	P(g_4_2T-T)	F4TCNQ	72.3 S/cm	[96]
Sequential Solution doping	Aligned PBTTT	F6TCNNQ	2400 S/cm	[44]
	PIDF-BT	F4TCNQ	~210 S/cm	[52]
	P3HT	F4TCNQ	~5.5 S/cm	[71]
	PIDF-BSe	F4TCNQ	~180 S/cm	[89]
	PBDTTT:EFT	F4TCNQ	~1.9 S/cm	[90]
	P(g_3_2T-OTz)	F4TCNQ	550 S/cm	[92]
	P(g_3_2T-Se)	F4TCNQ	1136 S/cm	[93]
	PCPDTSBT-A	F4TCNQ	1.27 S/cm	[95]
	PIDF-BTO4	F4TCNQ	1982 S/cm	[98]
Sequential vapor doping	PBTTT-C12	F4TCNQ	220 S/cm	[47]
	PBTTT-C14	F4TCNQ	~120 S/cm	[80]
	P3HT	F4TCNQ	4.2 S/cm	[81]
	PBTTT-C16	F4TCNQ	248 S/cm	[82]
Hybrid doping	PIDF-BT	F4TCNQ	~634 S/cm	[87]
	P3HT	F4TCNQ	~71 S/cm	[87]

## Data Availability

No new data were created or analyzed in this study. Data sharing is not applicable to this article.
